# Can Broadly Neutralizing HIV-1 Antibodies Help Achieve an ART-Free Remission?

**DOI:** 10.3389/fimmu.2021.710044

**Published:** 2021-07-12

**Authors:** Denise C. Hsu, John W. Mellors, Sandhya Vasan

**Affiliations:** ^1^ U.S. Military HIV Research Program, Walter Reed Army Institute of Research, Silver Spring, MD, United States; ^2^ Henry M. Jackson Foundation for the Advancement of Military Medicine, Bethesda, MD, United States; ^3^ Division of Infectious Diseases, Department of Medicine, University of Pittsburgh, Pittsburgh, PA, United States

**Keywords:** broadly neutralizing HIV-1 antibody, HIV remission, HIV cure, HIV immunotherapy, HIV therapeutics

## Abstract

Many broadly neutralizing antibodies (bnAbs) targeting the HIV-1 envelope glycoprotein are being assessed in clinical trials as strategies for HIV-1 prevention, treatment, and antiretroviral-free remission. BnAbs can neutralize HIV-1 and target infected cells for elimination. Concerns about HIV-1 resistance to single bnAbs have led to studies of bnAb combinations with non-overlapping resistance profiles. This review focuses on the potential for bnAbs to induce HIV-1 remission, either alone or in combination with latency reversing agents, therapeutic vaccines or other novel therapeutics. Key topics include preliminary activity of bnAbs in preclinical models and in human studies of HIV-1 remission, clinical trial designs, and antibody design strategies to optimize pharmacokinetics, coverage of rebound-competent virus, and enhancement of cellular immune functions.

## Introduction

Antiretroviral therapy (ART) has dramatically reduced the morbidity and mortality associated with human immunodeficiency virus type-1 (HIV-1) infection by suppressing viral replication (1, 2) but ART does not cure HIV-1 because of long-lived cells carrying replication-competent (intact) proviruses (3–5). Viral rebound occurs within weeks in most people with HIV-1 (PWH) who discontinue ART, including those who initiate ART early during acute infection with long-term successful suppression of plasma viremia measured as HIV-1 RNA (6, 7). Additionally, there are barriers to universal ART uptake that include toxicities, stigma, and the need for lifelong adherence (8–10). Therefore, alternative ART-free strategies that confer durable viral suppression, prevent disease progression, and avoid drug resistance are highly desirable (11, 12). Proposed minimal target profiles for these strategies include the ability to maintain plasma HIV-1 RNA below the level at which transmission occurs, for at least 2 years, and be generally safe and tolerated (13).

Broadly neutralizing antibodies target specific vulnerable sites on the HIV-1 envelope, mediate neutralization and target infected cells for elimination. In this review, we will focus on the potential for bnAbs to induce antiretroviral-free HIV-1 control, either alone or in combination with latency reversing agents, immune activating agents, therapeutic vaccines or other novel therapeutics.

## Broadly Neutralizing Antibodies in Clinical Development

**Figure 1:**
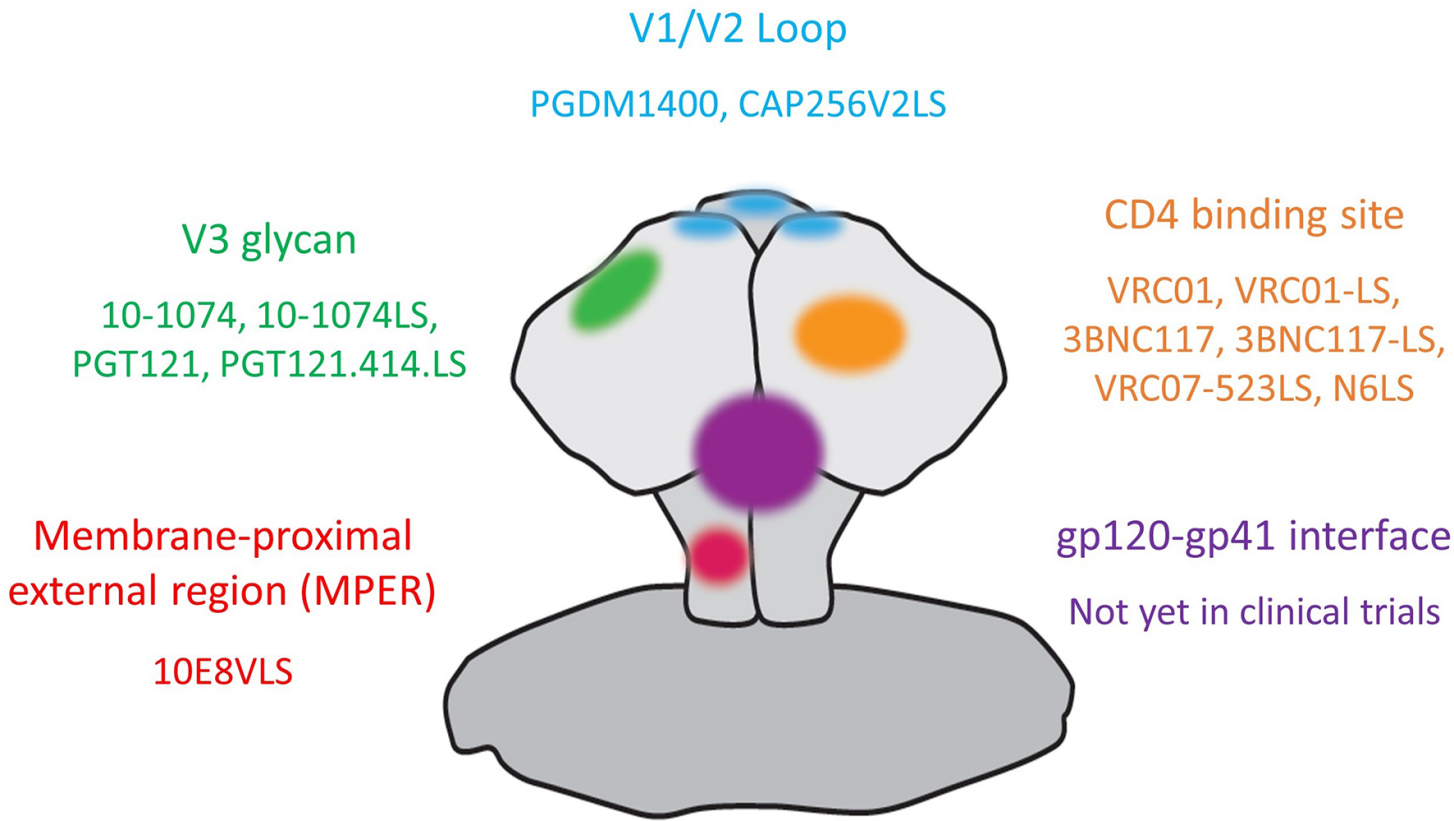
Anti-HIV-1 broadly neutralizing antibodies in clinical trials and their targets on the HIV-1 envelope.

Multiple bnAbs that exhibit breath and potency against epitopes on the HIV-1 envelope trimer are currently being assessed in clinical trials for HIV-1 prevention, treatment as well as remission induction. The targeted areas on HIV-1 envelope (see **Figure 1**) include the CD4-binding site (CD4bs) on gp120 [VRC01 (14); VRC01-LS (15), 3BNC117 (16), 3BNC117-LS (17), VRC07-523LS (18) and N6LS (19)]; the glycan-dependent epitopes on V1/V2 (PGDM1400 (20) and CAP256V2LS (21) as well as V3 loops (10-1074 (22), 10-1074-LS (17), PGT121 (23) and PGT121.414.LS); the linear epitopes in the membrane-proximal external region (MPER) on gp41 [10E8VLS (24, 25)]. Other bnAbs with high potency and breadth that have not yet entered clinical trials include antibodies targeting the gp120-gp41 interface (26) and the N49 lineage of CD4bs bnAbs (27, 28).

A number of clinical trials have shown that bnAbs (including VRC01, 3BNC117, 10-1074, VRC01-LS, VRC07-523LS, PGT121 and N6LS) are safe and well tolerated (29–38). Serious adverse events are rare but have been reported. A phase 1 study evaluating the subcutaneous administration of 10E8VLS alone or concurrently with VRC07-523LS in healthy adults was paused and the administration of 10E8VLS terminated due to local reactogenicity. Seven of 8 recipients of 10E8VLS experienced erythema and induration within 24 hours and a biopsy from 1 participant with induration demonstrated panniculitis (39).

The utility of bnAbs for HIV prevention have been reviewed extensively (40–42) and thus will not be a focus of this article. The recently completed Antibody Mediated Protection (AMP) trials, 2 parallel phase 2b multicenter, randomized, double-blind, placebo-controlled trials involving over 4600 participants and over 3000 recipients of VRC01, every 8 weeks for 20 months have further demonstrated the safety, feasibility and scalability of intravenous bnAb infusion (43). Though VRC01 did not prevent overall HIV-1 acquisition, it did reduce the acquisition of viruses highly sensitive (an 80% inhibitory concentration, IC80 of <1 μg/mL) to VRC01, emphasizing the importance of breath and potency for bnAb efficacy.

In PWH and plasma viremia, bnAb monotherapy leads to transient reductions in HIV-1 RNA of ~1.5 log_10_ copies/mL (in the absence of pre-existing resistance), with mean HIV-1 RNA reductions of 1.48, 1.14, 1.52 and 1.7 log_10_ copies/mL for 3BNC117, VRC-01, 10-1074 and PGT121, respectively (30–32, 36). These safety and antiviral activity data supported the investigation of bnAbs as potential therapeutics for HIV-1. The two main therapeutic applications that are in clinical evaluations are the use of bnAbs in place of current antiretroviral regimens to maintain viral suppression and the use of bnAb as part of a therapeutic combination to target and eliminate the HIV-1 reservoir.

## The Use of bnAb in Place of ART to Maintain Viral Suppression

The potential of bnAbs in maintaining viral suppression during analytical treatment interruption (ATI) has been assessed in a number of clinical trials. When 3BNC117 was administered during ATI to PWH with undetectable plasma HIV-1 RNA while on ART and with 3BNC117-sensitive outgrowth viruses, viral rebound was delayed to 8.4 weeks when compared to 2.6 weeks in matched historical controls. In the majority of participants, emerging viruses showed 3BNC117 resistance, indicating bnAb selection of escape variants. However, 30% of participants remained suppressed until 3BNC117 levels waned below 20 μg/mL, and the viruses emerging in all but one of these participants showed no apparent resistance to 3BCN117, suggesting failure of bnAb escape over a period of 9-19 weeks (44). In contrast, when 3BNC117 was administered to PWH at 24 weeks, 12 weeks and 2 days before ATI and 3 weeks after ATI, without pre-selection for 3NBC117 sensitivity, the time to rebound was strongly influenced by the neutralization sensitivity of the pre-treatment viruses, 3.6 vs 9.2 weeks in those with resistant vs sensitive viruses, respectively (45).

In 2 single arm clinical trials, AIDS Clinical Trials Group (ACTG) A5340, and National Institutes of Health (NIH) 15-I-0140, VRC01 during ATI was associated with a higher proportion of participants with undetectable HIV-1 RNA at 4 weeks post ATI when compared with historical controls. However, the difference was no longer significant at 8 weeks. VRC01 exerted pressure on the rebounding viruses, resulting in selection for preexisting and emerging bnAb-resistant viruses (46).

The aforementioned studies involved participants with treated chronic HIV-1 infection. PWH who initiated ART during acute HIV-1 infection (AHI) have smaller (47–49) and less diverse viral reservoirs (50, 51) as well as more preserved immune responses and thus may potentially display more favorable responses to bnAbs. VRC01 infusions during ATI in PWH who initiated ART during AHI did not significantly delay time to viral rebound of >20 copies/mL but did delay time to viral rebound of >1000 copies/mL, at a median of 33 days in VRC01 recipients when compared to 14 days in placebo recipients (52). Importantly, neutralization sensitivity was similar after viral rebound when compared to at the time of AHI diagnosis, indicating the lack of selection for VRC01 resistance during ATI. This was most likely secondary to the near-absence of diversity among these participants’ sequences and short duration of viral replication due to prompt ART initiation. Consistent with studies in chronic HIV-1 infection, participants with strains most sensitive to VRC01 rebounded later. Interestingly, viral rebound occurred while the average serum VRC01 level was 50 times higher than *in vitro* IC80 values, suggesting that the VRC01 concentrations achieved were therapeutically insufficient. This may be a result of inadequate levels or speed of VRC01 penetration into tissue reservoirs, off target protein binding, or other factors, emphasizing that *in vitro* IC50 orIC80 values do not necessarily translate to therapeutic concentrations in humans (53).

Collectively, these clinical trials indicate that bnAb monotherapy does not have sufficient breadth to prevent rebound in most individuals. However, in the setting of adequate neutralization breadth to cover rebound competent viruses, bnAbs have the potential to maintain viral suppression during ATI as long as therapeutic levels are maintained in plasma and tissues, although further studies are needed to define adequate therapeutic levels.

## Mechanisms to Improve Potential for bnAbs to Suppress Viral Replication

### Increasing Half-Life and Potency

The current requirement for frequent dosing (monthly) reduces the appeal of bnAbs to maintain ART-free viral suppression. Thus, two amino acid mutations (methionine-to-leucine substitution and an asparagine-to-serine substitution at amino acid positions 428 and 434, respectively, collectively referred to as “LS”) have been introduced into the fragment crystallizable (Fc)-region of a number of bnAbs to improve affinity to the neonatal Fc-receptor (FcRn), leading to recirculation following cellular endocytosis and thereby extending the *in vivo* half-life (15, 17, 54).

In non-human primates (NHP), the LS substitution extended serum half-life by 2-3 fold (17) and also resulted in longer periods of protection against repeated mucosal challenges with Simian/Human Immunodeficiency Virus (SHIV), expressing HIV-1 envelope on a SIV backbone, with medians of 14.5 vs 8, 27 vs 12.5 and 17 vs 13 weeks for VRC01-LS, 10-1074-LS and 3BC117-LS, respectively when compared to the unmodified parental bnAb (17, 55). In people without HIV, the LS substitution extended serum half-life of VRC01 from 15 to 71 days (34). Serum half-life after intravenous infusion was 38 days for VRC07-523LS (35) and 44 days for N6LS (38). Pharmacokinetics data for 3BNC117-LS (NCT03254277), 10-1074LS (NCT03554408), PGT121.414.LS (NCT04212091) and CAP256V2LS (NCT04408963) are forthcoming, and the impact of LS mutations on bnAb half-life in tissues is yet to be quantified.

Other mutations to extend half-life have also been reported, including the Met252Tyr, Ser254Thr, and Thr256Glu (YTE) substitution that is associated with a 4-fold increase in serum half-life (56, 57). However, YTE substitution also reduces ADCC activity of the antibody (56), which potentially reduces its utility in HIV remission induction strategies that will likely require ADCC.

Subcutaneous administration of bnAb by direct needle and syringe injection obviates the need for venous access and volumetric pump infusion and is thus more scalable for widespread use. However, when compared with intravenous infusion, subcutaneous injections of VRC01, VRC01-LS, VRC07-523LS and N6LS showed markedly reduced maximal serum concentration (C_max_) and delayed time to maximal concentration T_MAX_ (29, 34, 35, 38).

Next-generation sequencing, computational bioinformatics, and structure-guided design can be employed to modify bnAbs to enhance neutralization potency and breadth. This approach was applied to VRC01 and resulted in the development of VRC07-523-LS, that is over 5-fold more potent than VRC01 and neutralized 96% of a panel of 171 HIV-1 pseudotyped viruses *in vitro* (18).

### Addressing the Issue of bnAb Activity, Breadth and Resistance

Certain HIV-1 strains are intrinsically resistant to bnAbs targeting specific epitopes. BnAbs targeting V3 glycans, including 10-1074 and PGT121, have little to no neutralizing activity against CRF01_AE and can only neutralize a minority of Clade D strains (22). In contrast, bnAbs targeting the V1/V2 loop have suboptimal activity against Clade B strains, with PGDM1400 neutralizing 70% (20) and CAP256-VRC26.25 neutralizing only 15% (58). Thus, data regarding the major prevalent subtypes for a particular geographic location must be considered in the selection of bnAbs.

In PWH with plasma viremia, bnAb mediated HIV-1 RNA reductions were mostly observed in those with sensitive HIV-1 strains. Furthermore, reduction in sensitivity developed within weeks of monotherapy, due to expansion of pre-existing resistant viruses or selection of new resistant variants (30–32, 36). Screening for pre-existing resistant variants is especially relevant for clinical trials assessing the utility of bnAbs during ATI. BnAb sensitivity of virus in plasma can be readily determined in participants with viremia; whereas in participants on suppressive ART, bnAb sensitivity can be determined using HIV-1 enveloped pseudovirus derived from proviral DNA in PBMC or directly using viruses from outgrowth cultures (31, 44, 59). However, these assays are labor intensive, impractical for widespread implementation, and may not capture the spectrum of minor viral variants that could emerge (60, 61).

An approach to expand neutralization coverage is to use bnAbs targeting different HIV-1 envelope epitopes in combination. Combinations of three and four bnAbs can neutralize 98-100% of viruses from a diverse panel of 125 Env-pseudotyped viruses *in vitro* (62, 63). In clinical trials, the combination of 3BNC117 and 10–1074 was safe and generally well tolerated and pharmacokinetics were similar to when these bnAbs were administered as monotherapy (61, 64).

Co-administration of 3BNC117 and 10–1074 to PWH and plasma viremia resulted in an average 1.65 log_10_copies/mL reduction in HIV-1 RNA. The four participants with dual antibody-sensitive viruses had greater and more prolonged HIV-1 RNA reduction (average of 2.05 log_10_ copies/mL for over 3 months). Suppression to undetectable levels was seen in one participant with low (730 copies/mL) pre-bnAb HIV-1 RNA. In 3 of the 4 initially sensitive participants, rebound viruses were resistant to 10–1074, but remained sensitive to 3BNC117 (as the shorter half-life of 3BNC117 resulted in a tail of 10–1074 monotherapy) (60). Thus, in PWH with plasma viremia, dual bnAbs are not sufficient to suppress viremia to undetectable levels.

In contrast, co-administration of 3BNC117 and 10–1074 during ATI to PWH who had undetectable plasma HIV-1 RNA while on ART and outgrowth viruses that were sensitivity to both 3BNC117 and 10-1074, maintained viral suppression for extended durations. The median time to rebound was 21 weeks compared to 8.4 weeks with 3BNC117 monotherapy and 2.3 weeks for historical controls. In participants with no detectable resistant viruses pre-infusion, viral rebound occurred when the levels of bnAb waned. Rebound viruses were resistant to 10–1074, but remained sensitive to 3BNC117 (due to the shorter half-life of 3BNC117) but none developed viruses resistant to both antibodies (61). This study supports that bnAbs used in combination can maintain long-term viral suppression in PWH with antibody-sensitive viruses as long as therapeutic levels are maintained. A number of clinical trials assessing the efficacy of combinations of bnAbs in maintaining viral suppression during ATI are currently ongoing (**Table 1**). Differential clearance of bnAbs used in combination that could result in a tail of bnAb monotherapy remains an issue to be addressed by thorough understanding of bnAb pharmacokinetics and interactions in clinical trials.

**Table 1:** Ongoing clinical trials of bnAb as part of HIV-1 therapeutic or remission strategy.

BnAb	Target site	Additional Intervention(s)	Study population	Study Endpoints	Clinicaltrials.gov identifier
**Studies on antiviral effects of bnAbs**					
VRC01-LS or VRC07-523LS	CD4bs		Adults with HIV-1 and plasma viremia	Safety, PK and effects on plasma viremia	NCT02840474
3BNC117 + 10-1074	CD4bs, V3 glycans		Adults with HIV-1 and plasma viremia	Safety and effects on plasma viremia	NCT03571204
3BNC117-LS + 10-1074-LS	CD4bs, V3 glycans		Adults with HIV-1 and plasma viremia	Safety, PK and effects on plasma viremia	NCT04250636
VRC07-523LS + PGT121 + PGDM1400	CD4bs, V3 glycans, V1/V2 loop		Adults with HIV-1 and plasma viremia	Safety, PK and effects on plasma viremia	NCT03205917
10E8.4/iMab	MPER, CD4		Adults with HIV-1 and plasma viremia	Safety, PK and effects on plasma viremia	NCT03875209
SAR441236	Trispecific Ab targeting CD4bs, V2 loop, MPER		Adults with HIV-1 and plasma viremia	Safety, PK and effects on plasma viremia	NCT03705169
VRC01	CD4bs		Adults at the diagnosis of acute HIV-1 infection, in addition to ART	Safety and effects on plasma viremia	NCT02591420
VRC01	CD4bs		Infants with HIV-1, in addition to ART	Safety, PK and effects on plasma viremia	NCT03208231
3BNC117	CD4bs	Albuvirtide (Fusion inhibitor)	Adults with multi-drug resistant HIV-1	Effects on plasma viremia	NCT04560569
**Studies on efficacy in maintaining viral suppression during ATI**					
3BNC117 + 10-1074	CD4bs, V3 glycans		Adults with HIV-1, on ART	Effects on the latent reservoir, impact on viral rebound and safety	NCT03526848
3BNC117 + 10-1074	CD4bs, V3 glycans		Adults with HIV-1, on ART, initiated during primary HIV-1 infection	Safety and impact on viral rebound	NCT03571204
VRC01-LS + 10-1074	CD4bs, V3 glycans		Children with antepartum or peripartum HIV-1 infection, on ART, initiated early after diagnosis	Safety, impact on viral rebound and PK	NCT03707977
VRC01 + 10-1074	CD4bs, V3 glycans		Adults with HIV-1, on ART	Safety and impact on viral rebound	NCT03831945
3BNC117-LS + 10-1074-LS	CD4bs, V3 glycans		Adults with HIV-1, on ART, initiated during primary HIV-1 infection	Safety and impact on viral rebound	NCT04319367
VRC07-523LS + PGT121 + PGDM1400	CD4bs, V3 glycans, V1/V2 loop		Adults with HIV-1, on ART	Safety, PK and impact on viral rebound	NCT03721510
3BNC117	CD4bs	Albuvirtide (Fusion inhibitor)	Adults with HIV-1, on ART	Impact on viral rebound	NCT03719664
3BNC117-LS + 10-1074-LS	CD4bs, V3 glycans	Lenacapavir (capsid inhibitor)	Adults with HIV-1, on ART	Safety, impact on viral rebound and PK	NCT04811040
VRC07-523LS	CD4bs	Long-acting cabotegravir	Adults with HIV-1, on ART	Safety and impact on viral rebound and PK	NCT03739996
**Studies on bnAbs in combination with additional interventions to target and eliminate the viral reservoir**					
3BNC117	CD4bs	Romidepsin (LRA)	Adults at the diagnosis of HIV-1 infection, in addition to ART	Safety, effects on plasma viremia and the latent reservoir	NCT03041012
3BNC117	CD4bs	Romidepsin	Adults with HIV-1, on ART	Safety, impacts on viral rebound and the latent reservoir	NCT02850016
VRC07-523LS	CD4bs	Vorinostat (LRA)	Adults with HIV-1, on ART	Safety, effects on viral reservoir	NCT03803605
3BNC117 + 10-1074	CD4bs, V3 glycans	Lefitolimod (TLR9 agonist)	Adults with HIV-1, on ART	Safety and impact on viral rebound	NCT03837756
VRC07-523LS + 10-1074	CD4bs, V3 glycans	N-803 (IL-15 superagonist)	Adults with HIV-1, on ART	Safety, impacts on viral rebound and the latent reservoir, PK	NCT04340596
3BNC117 + 10-1074	CD4bs, V3 glycans	Pegylated Interferon alpha 2b	Adults with HIV-1, on ART	Safety, NK cell activity and impact on viral rebound	NCT03588715
VRC07-523LS + 10-1074	CD4bs, V3 glycans	HIV DNA vaccine, HIV MVA vaccine, lefitolimod	Adults with HIV-1, on ART	Safety, proportion with post treatment control, immunogenicity	NCT04357821
10-1074	V3 glycans	HIV RNA vaccine, romidepsin	Adults with HIV-1, on ART	Safety, impacts on viral rebound and the latent viral reservoir, immunogenicity	NCT03619278

### Expanding bnAb Breadth

Newer technologies allow for the construction of antibodies with two, three, or four different binding sites on a single molecule. Targeting multiple HIV-1 envelope epitopes in a single bnAb molecule reduces the risk of developing viral resistance during periods of essential monotherapy due to differential half-lives of combination bnAbs, simplifies manufacturing and downstream product development and thus scalability. The 10E8V2.0/ibalizumab bispecific ab, generated using CrossMAbs technology, neutralized 100% of a panel of 118 HIV-1 pseudotyped viruses *in vitro* with mean IC50 values of 0.002 μg/mL. Furthermore, it also protected humanized mice against repeated intraperitoneal HIV-1_JR-CSF_ challenges (65). Currently, a phase I dose-escalation study on the safety, tolerability, pharmacokinetics, and anti-HIV-1 activity of the 10E8.4/ibalizumab bispecific Ab is in progress (NCT03875209).

Tri-specific abs have also been generated using knob-in-hole heterodimerization to pair a single arm derived from a normal immunoglobulin (IgG) with a double-arm generated in the cross-over dual variable Ig-like proteins (CODV-Ig). Two lead tri-specific Ab, N6/PGDM1400-10E8v4 and VRC01/PGDM1400-10E8v4 were able to neutralize 207 and 204 of 208 pseudotyped viruses *in vitro*, respectively. When NHP were challenged intrarectally with a mix of SHIV_325c_ (resistant to VRC01) and SHIV_BaLP4_ (resistant to PGDM1400), 6 of 8 animals infused with VRC01 alone and 5 of 8 animals infused with PGDM1400 became infected. In contrast, none of the 8 animals infused with VRC01/PGDM1400-10E8v4 trispecific Ab were infected (66). This demonstrated that tri-specific ab conferred protection against viruses that otherwise showed resistance to single parental bnAbs. The VRC01-LS/PGDM1400-10E8v4 trispecific ab (SAR441236) is currently being evaluated in a dose escalation study to determine safety, pharmacokinetics and anti-HIV-1 activity (NCT03705169).

It is anticipated that the modified versions of 10E8 used in the aforementioned bi- and tri-specific bnAbs will not recapitulate the local reactogenicity associated with 10E8VLS due to differences in amino acid sequences as well as antibody-antigen interactions between the bivalent 10E8VLS and the monovalent 10E8.4 arm of the bi- or tri-specific bnAbs. Due to their novel structures, recombinant multi-specific Abs have a higher theoretical possibility of inducing anti-drug Abs. However, this has not been reported in the preliminary trials to date and will need to be confirmed in larger scale studies in humans.

## The Use of bnAbs to Target, Control and Potentially Eliminate the Viral Reservoir

### BnAbs Alone

Data from rhesus macaques inoculated with SHIV suggest that bnAb when administered alone may induce sustained ART free remission (**Table 2**). When 3BNC117 and 10-1074 were administered to animals 3 days post intra-rectal SHIV_AD8-EO_ inoculation, plasma viremia was only detectable in 2 of 6 animals in the first month. Viral suppression was maintained for 8-25 weeks until bnAb levels waned. Importantly, 3 animals developed viral control. In a follow-on experiment, the same bnAb regimen was administered 3 days post intravenous SHIV_AD8-EO_ inoculation. Plasma viremia was initially detected in all animals post inoculation (PI), but then declined to undetectable levels by 4 weeks post bnAb administration. Rebound occurred at 7-16 weeks PI when bnAb levels waned. Three of 7 animals developed post-rebound viral control. Subsequent CD8 T cell depletion in all 6 controllers resulted in increase in plasma viremia, suggesting a CD8 T cell dependent mechanism for control (67).

**Table 2:** Non-human primate studies on the use of bnAbs to target, control and potentially eliminate the viral reservoir.

	SHIV	ART initiation	BnAb	Additional Interventions	Outcome
Nishimura et al. (67)	SHIV_AD8-EO_	No ART	3BNC117 + 10-1074 at days 3, 10 and 17 PI		Viral control in 3 of 6 animals from intra-rectal and 3 of 7 animals from intravenous inoculation
Nishimura et al. (68)	SHIV_AD8-EO_	2 weeks post infection	3BNC117 + 10-1074 alone at weeks 2, 4 and 6 PI or ART initiation at week 2; 3BNC117 + 10-1074 at weeks 9, 11 and 13; ART discontinuation at week 10		Viral control in 4 of 6 animals in bnAb alone arm in 3 of 6 animals in bnAb and ART arm
Borducchi et al. (69)	SHIV_SF162P3_	7 days post infection	PGT121	TLR7 agonist	No rebound in 5/11 animals and delay in rebound when compared to controls (112 vs 21 days)
Hsu et al. (70)	SHIV_1157ipd3N4_	2 weeks post infection	PGT121 + N6-LS	TLR7 agonist	Delay in rebound when compared to controls (6 versus 3 weeks)
Barouch et al. (71)	SHIV_SF162P3_	12 months post infection	PGT121 or its FC modified version (GS9721)	TLR7 agonist	No rebound in 7/17 animals
Whitney et al. (72)	SHIV_AD8_	~50 days post infection	3BNC117 + 10-1074	IL-15 superagonist	No difference in time to rebound. Post-rebound control in 6 of 8 animals
Barouch et al. (73)	SHIV_SF162P3_	9 days post infection	PGT121	TLR7 agonist, Ad26/MVA vaccine	4 of 12 PGT121+TLR7 agonisttreated animals and 4 of 10 Ad26/MVA vaccine+PGT121+TLR7 agonist-treated animals did not rebound post treatment interruption. Only 4 of 10 Ad26/MVA vaccine+PGT121+TLR7 agonist-treated animals remained viremic 140 days post treatment interruption.

To evaluate the above strategy at a more clinically relevant post infection timepoint, SHIV_AD8-EO_ -infected monkeys were treated with bnAbs (10-1074 and 3BNC117) alone or with ART plus bnAbs starting at 2 weeks PI. In the bnAb alone group, bnAbs reduced plasma viremia overall, but only 1 animal achieved full plasma viral suppression. This animal later rebounded and subsequently regained control. Three other animals also controlled virus, but much later, at 90-150 weeks post infection. Viral rebound occurred in all animals in the ART plus bnAbs group when bnAb levels waned. Three animals developed post-rebound viral control at weeks 70-160. CD8 T cell depletion in controllers from both groups also led to transient increases in plasma viremia (68).

Therefore, bnAbs initiated on day 3 or day 14 PI resulted in CD8 T cells dependent viral control in about half the animals. However, the time required to develop viral control was much more protracted when the initiation of bnAbs were delayed. The authors speculated that though bnAbs administered early post infection suppressed viremia, very-low levels of antigen production likely persisted and in the presence of bnAbs stimulated immune complex formation and dendritic cell activation leading to the induction of CD8 T cell responses. Immune responses were further augmented during viral rebound, culminating in viral control.

There is little data in humans on the efficacy of bnAbs to induce T cell responses leading to viral control. In the study by Mendoza et al, in which a combination of 3NBC117 and 10-1074 was administered during ATI, increased Gag-specific CD8+ and CD4+ T cell responses were seen in 9/9 and 8/9 participants with sensitive viruses and prolonged viral suppression. The increases were attributed to both newly detectable reactivity to HIV-1 Gag epitopes and the expansion of pre-existing measurable responses (74). However, whether the increase in responses contribute to viral control remains to be elucidated.

Data from human trials involving bnAbs infusions concurrent with ART [including VRC01 (31, 75) and 3BNC117 (45)] showed no measurable impact on the latent reservoir. The impact of bnAbs on the reservoir would likely be improved when used in combination with other strategies, including latency reversal agents to induce proviral activation and cell-surface expression of viral envelopes so that they can be targeted by bnAbs, immune activating agents to enhance anti-viral responses and Fc-mediated killing of infected cells, or therapeutic vaccination to stimulate T cell responses for viral control.

### BnAbs and Immune Activating Agents

In the study by Borducchi et al., toll-like receptor 7 (TLR7) agonist was incorporated to induce innate immune activation and enhance anti-viral responses (76, 77). In this study, TLR7 agonist and PGT121 were administered in addition to ART (initiated at 7 days post SHIV_SF162P3_ inoculation). ART was discontinued after antibody washout at week 130. Only 6 of 11 (55%) animals that received PGT121+TLR7 agonist rebounded, at a median of 112 days vs 21 days in controls (p=0.0001) (69). Interestingly, no induction of CD8 T cell responses was seen. Importantly, in the animals that did not rebound, adoptive transfer experiments did not reveal infection of naïve hosts. Furthermore, SHIV RNA also remained undetectable after CD8 T cell depletion. These data suggest that the combination of immune stimulation with bnAb administration may potentially eliminate the viral reservoir.

In a follow-on study by Hsu et al., rhesus macaques inoculated with SHIV_1157ipd3N4_ were initiated on ART on day 14 to more closely mirror what is logistically feasible in humans. ART initiation was followed by the administration of TLR7 agonist and dual bnAbs (N6-LS and PGT121). ART was discontinued after antibody washout. Though TLR7 agonist and dual bnAbs delayed viral rebound by 2-fold (3 vs 6 wks, p=0.024), viral rebound occurred in all animals (70). The delay in ART initiation, the shorter duration of ART and the lower number of doses of bnAbs administered (ranging from 2-5 doses, limited by the development of anti-drug antibody) may have contributed to the reduction in efficacy when compared with the Borducchi et al. study.

In a recent study, Barouch et al., demonstrated in SHIV_SF162P3_-infected animals that initiated ART 12 months after infection, TLR7 agonist and PGT121 or its Fc-modified version, GS-9721 prevented viral rebound in 7 of 17 animals following ART discontinuation. These data suggest that TLR7 agonist and bnAb administration is efficacious for ART-free remission of chronic SHIV infection (71).

Whitney et al. explored the use of N-803 [IL-15 superagonist that has been shown to increase NK and CD8 T cells in the peripheral blood as well as SHIV-specific CD8 T cells in lymphoid follicles (78–80)] in combination with 3BNC117 and 10-1074 in macaques on ART for chronic SHIV_AD8_ infection. ATI occurred after bnAb washout. Viral rebound occurred in all animals and no differences in time to rebound were seen between the active and control groups. However, post-rebound viral control occurred in 6 of 8 animals in the active group 4 months from the start of ATI (72).

Data regarding the effects of bnAbs in combination with additional strategies on the latent reservoir in humans is forthcoming. In a trial of 3BNC117 and romidepsin [a histone deacetylase inhibitor (81)], in 20 PWH on ART, the addition of 3BNC117 did not significantly reduce HIV-1 DNA or delay viral rebound when compared to romidepsin alone (82). A number of clinical trials, including 3BNC117+romidepsin (NCT03041012, NCT02850016), VRC07-523LS+vorinostat (NCT03803605), pegylated-interferon Alpha 2b+3BNC117+10-1074 (NCT03588715), lefitolimod (TLR9 agonist)+3BNC117+10-1074 (NCT03837756) and N-803+VRC07-523LS+10-1074 (NCT04340596) are currently on-going (**Table 1**).

### Use of bNabs With Vaccination

The effects of immune stimulation (TLR agonism) and bnAbs may be further enhanced by the addition of therapeutic vaccine to induce anti-HIV-1 CD8 T cell responses. This strategy was explored in SHIV_SF162P3_-infected rhesus macaques that were initiated on ART day 9 post infection. Following ART discontinuation, all control animals rebounded. All 12 of the Ad26/MVA vaccine+TLR7 animals also rebounded, but 3 developed post-rebound viral control. In contrast, only 8 of 12 of PGT121+TLR7 treated animals and 6 of 10 of Ad26/MVA vaccine+PGT121+TLR7 treated animals rebounded. Moreover, some animals exhibited post-rebound viral control so that by day 140 following ART discontinuation, only 4 of 10 of Ad26/MVA vaccine+PGT121+TLR7 treated animals have detectable viremia (73). Thus, combined TLR7 agonist, active and passive immunization resulted in both delayed viral rebound and post-rebound control following ART. This strategy is also being explored in an upcoming clinical trial involving therapeutic conserved element DNA vaccine+MVA vaccine+VRC07-523LS+10-1074+lefitolimod (NCT04357821) and ChAdOx/MVA HIV mosaic vaccine+3BNC117LS+10-1074LS+vesatolimod (ACTG5374) in PWH who initiated ART during acute HIV infection. Such human data is essential to determine whether results in NHP can be reproduced.

Data regarding the use of bnAbs to target, control and potentially eliminate the viral reservoir are largely from small NHP studies. However, there are differences between SHIV-infected NHP and PWH including lower viral diversity and higher rate of natural control in SHIV infection. Furthermore, efficacy of bnAbs is reduced with delay in bnAb administration or ART initiation, suggesting a narrow window of opportunity to intervene. Finally, a prolonged duration of viremia is also required for the observation of post treatment control. Thus, feasibility and translatability in PWH who started ART during chronic HIV infection is yet to be demonstrated.

## Incorporating Lessons Learned Into Clinical Trial Design

### The Use of bnAbs to Maintain Viral Suppression

Available data demonstrated that bnAbs are generally safe and suggest that bnAbs can maintain viral suppression during ATI as long as the pre-existing viruses are sensitive and therapeutic levels are maintained. Judicious selection of bnAbs based on neutralization-sensitivity of the predominant variants in a given geographic location, screening for pre-existing resistance and the use of bnAbs in combination to increase breadth and coverage will minimize the selection of escape variants. These optimization strategies will need to be further assessed in phase II or III clinical trials involving participants with chronic HIV infection to determine longer term efficacy. The ultimate goal is to identify antibody combinations with adequate breath to cover for circulating variants to obviate the need for screening for pre-existing resistance, which is a major barrier to widespread use. When bnAbs are used in combination in the absence of ART, considerations must be given to differences in half-life of each respective bnAb. In addition to peripheral blood, collection of tissue samples including lymph node and gut biopsies should be incorporated into clinical trials to allow measurement of the levels of bnAbs so as to inform what constitutes therapeutic levels in reservoir sites.

The use of bnAbs with LS modifications will extend duration above therapeutic threshold and reduce infusion frequency. This, together with alternative routes of administration such as subcutaneous injection will facilitate scale-up and access. Data from clinical trials evaluating long-acting injectable anti-HIV drugs, including monthly intramuscular cabotegravir (integrase strand transfer inhibitor) and rilpivirine (nonnucleoside reverse-transcriptase inhibitor) (83) or 6-monthly subcutaneous lenacapavir (capsid inhibitor) (84) are becoming available. Monthly intramuscular cabotegravir and rilpivirine demonstrated non-inferior viral suppression when compared to standard ART. The development of resistance is infrequent. However, injection-related adverse events were common (>80% of participants) but only infrequently led to medication withdrawal (83). Long acting ART will contend with bnAbs as preferred agents to maintain long-term viral suppression with infrequent dosing. Given that both strategies have pros and cons, usage and uptake will likely be driven by considerations including availability, local HIV-variant sensitivity profile, tolerability, relative cost and availability, and individual and local cultural preferences. While some may prefer a daily oral pill, others may prefer a less frequent schedule of administration. Dosage route, frequency, and potential for bnAbs self-administration will be important factors in this consideration.

### The Use of bnAbs to Induce HIV Remission

BnAbs alone are unlikely to be adequate to eliminate the latent reservoir. A handful of NHP studies where bnAbs were used in combination with innate immune activating agents have shown promise. However, mechanisms for delay in viral rebound or post-rebound viral control have not been delineated. The strategy of innate stimulation, active and passive immunization are in the early stages of development and data is needed to inform decisions regarding optimal timing and order of administration of individual interventions to maximize therapeutic effects.

It is important to bear in mind that immune activating agents may potentially expand the reservoir through clonal proliferation. It remains possible that certain reservoir sites including the central nervous system may be exposed to latency activation and viral replication but little bnAb mediated anti-HIV-1 effects due to the blood brain barrier limiting bnAb penetrance. These questions can be addressed in NHP models where direct tissue sampling of the brain is more feasible. In parallel, clinical trials administering bnAb therapies can also monitor differential impact on bnAb therapies administered by varying routes on viral burden or cellular reservoirs in the cerebrospinal fluid relative to blood.

Data from NHP studies suggest that the opportunity to intervene may be narrow, with reduction in efficacy or substantial increase in time required to observe results when bnAb administration or ART initiation is delayed just by a few weeks. The MHRP RV217 prospective cohort involving seronegative high-incidence populations who underwent twice-weekly HIV-1 RNA testing estimated the eclipse phase (the time between HIV-1 infection and a diagnosable infection by nucleic acid testing) to be one week (85). Therefore, taking into account the time required to diagnose, screen and then enroll participants into a clinical trial, the earliest that interventions can realistically be administered is likely around 2 weeks post infection. The clinical trial NCT02591420 that explores the effects of VRC01 when administered at the time of diagnosis of AHI on the viral reservoir has just completed clinical follow-up, demonstrating that intervening early during HIV-1 infection is logistically feasible. However, the majority of PWH initiated ART during chronic infection and the impacts of these strategies in this setting are yet to be defined.

Though NHP studies demonstrated that post-rebound viral control is possible, a protracted period of viremia may be required prior to the development of control. The delicate balance between the utility of an extended ATI to observe post-rebound viral control versus the associated risks of transmission to sexual partners, symptomatic HIV disease, immune depletion, and emergence of new drug resistance mutation has generated much debate among researchers, ethicists and PWH (86–89). Research in delineating mechanisms and/or correlates for delay in viral rebound and sustained post-rebound viral control is urgently needed to reduce the reliance on extended ATI as an outcome measure.

BnAbs are generally safe and well tolerated and have been shown to maintain viral suppression in the setting of sensitive pre-treatment viruses. Mechanisms to improve antiviral effects and ease of use are becoming available. Thus, bnAbs used in combination have the potential to replace ART and obviate the need for high level adherence that is necessary for daily ART. The use of bnAbs as a component of combination strategies to target the reservoir has shown promise in NHP models. However, the window of opportunity to intervene for maximal effect may be narrow. A remission strategy should be effective across the spectrum of HIV infection. Thus, much work needs to be done to answer questions regarding the penetration into tissue sites, what constitutes therapeutic levels, and the mechanisms of action in the delay of viral rebound and post-rebound control before bnAb can become an important therapeutic advance for PWH.

## Author Contributions

All authors reviewed the literature, drafted and revised the paper. All authors contributed to the article and approved the submitted version.

## Funding

SV reports funding from the U.S. Department of Defense with Henry M. Jackson Foundation for the Advancement of Military Medicine (Cooperative agreement numbers W81XWH-11-2-0174, W81XWH-07-2-0067, W81XWH-18-2-0040), funding from the NIH/NIAID (1UM1AI126603, 2UM1AI108568-08), and funding from the US Department of Defense (W81XWH1810579). DH reports funding from the U.S. Department of Defense with Henry M. Jackson Foundation for the Advancement of Military Medicine (Cooperative agreement number W81XWH-18-2-0040) and funding from the NIH/NIAID (1UM1AI126603, 2UM1AI108568-08). JM reports funding from NIH/NIAID to the I4C Martin Delaney Collaboratory (UM1AI126603), to the Pitt-Ohio State Clinical Trials Unit (UM1 AI069494), to the Pitt Virology Specialty Laboratory (UM1 AI106701), to the University of Pittsburgh (U01AI131285 and U01AI152969), from NCI through Leidos (75N91019D00024), and from the Bill & Melinda Gates Foundation (OPP1115715).

## Disclaimer

The U.S. Military HIV Research Program (MHRP) and the Emerging Infectious Diseases Branch (EIDB) at the Walter Reed Army Institute of Research are supported through a cooperative agreement with the Henry M. Jackson Foundation for the Advancement of Military Medicine (HJF). The views expressed are those of the authors and should not be construed to represent the positions of the U.S. Army, the Department of Defense or the Henry M. Jackson Foundation for the Advancement of Military Medicine, Inc.

## Conflict of Interest

SV and DH report grants from the U.S. Department of Defense with Henry M. Jackson Foundation for the Advancement of Military Medicine and grants from the NIH/NIAID for the submitted work. SV also report grants from the U.S. Department of Defense. JM reports grants from the NIH for the submitted work, and grants from Gilead Sciences, Janssen Pharmaceuticals, USAID, and personal fees from Accelevir Diagnostics, Gilead Sciences, Merck, Xi’an Yufan Biotechnologies, and other from Infectious Diseases Connect, Co-Crystal Pharmaceuticals, Inc., and Abound Bio, Inc., outside the submitted work.
